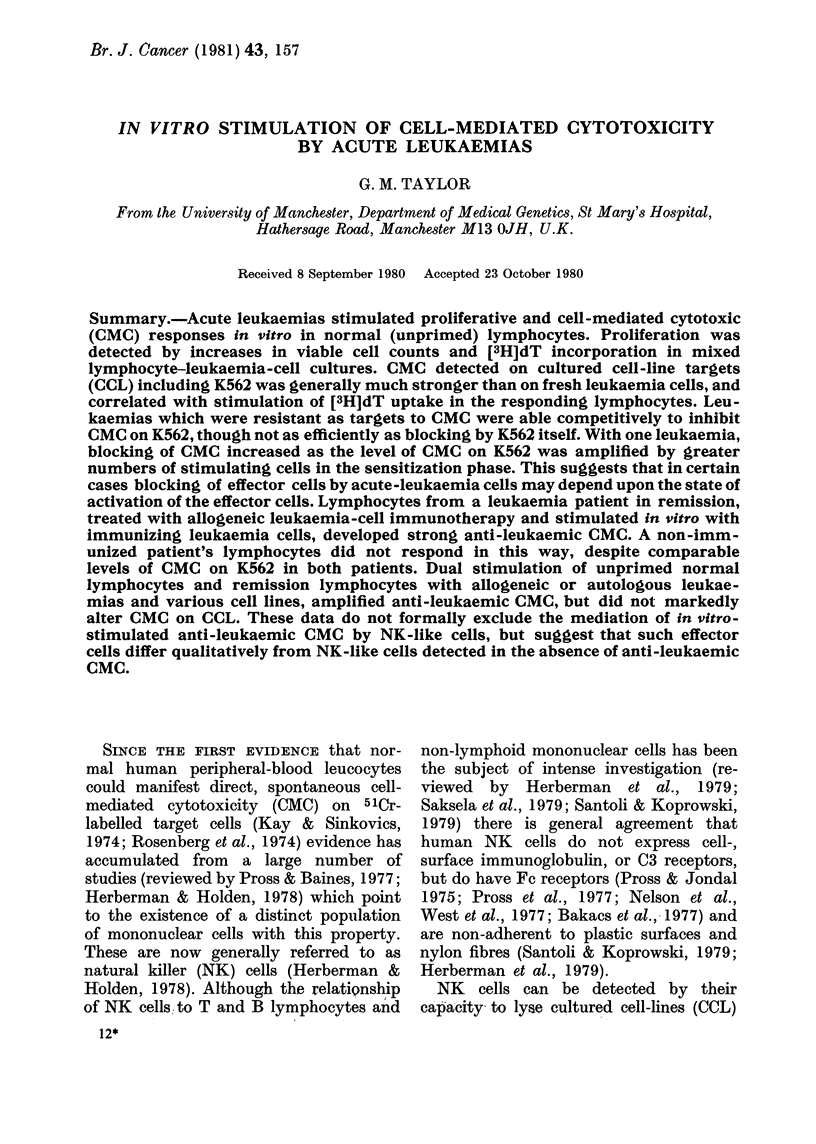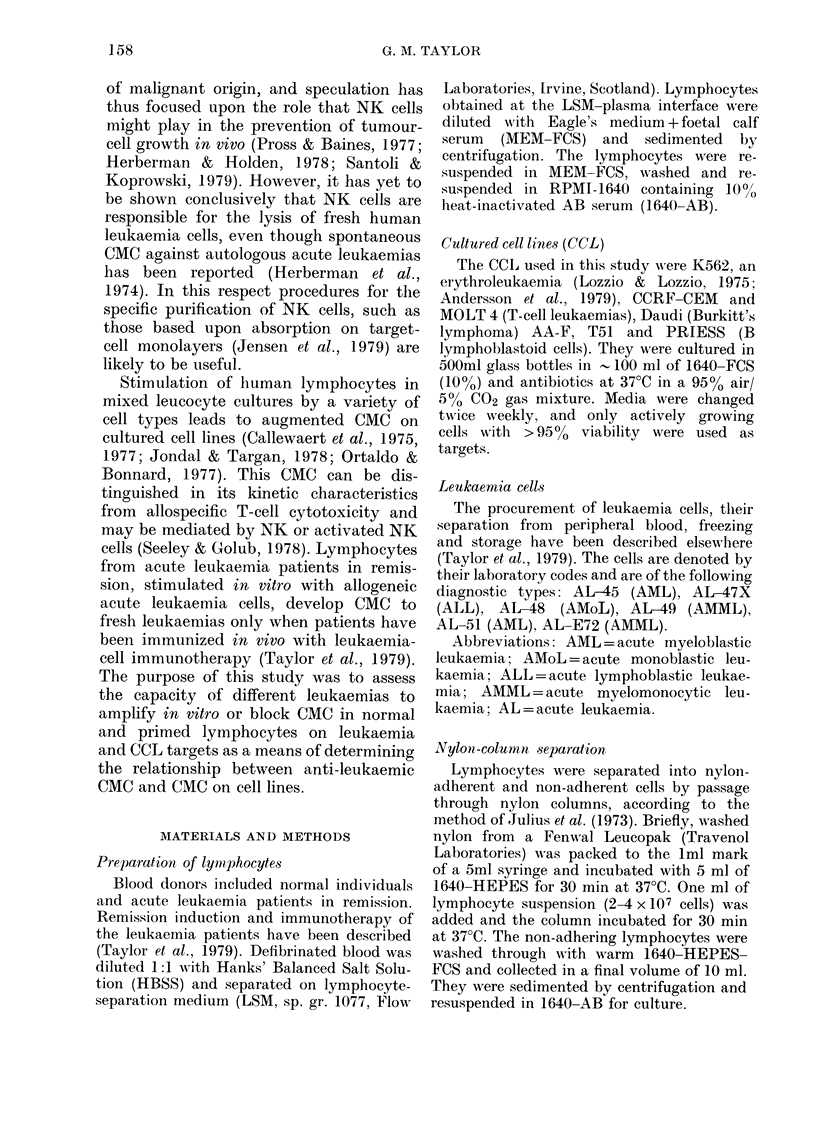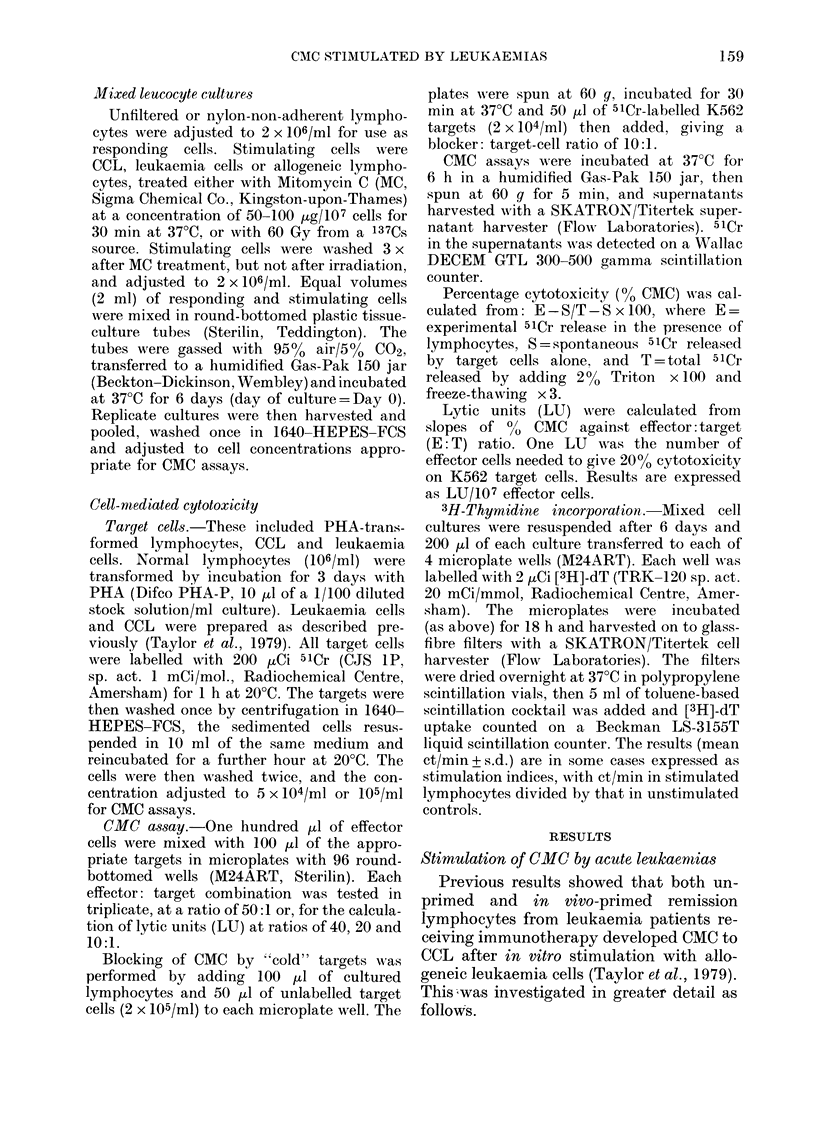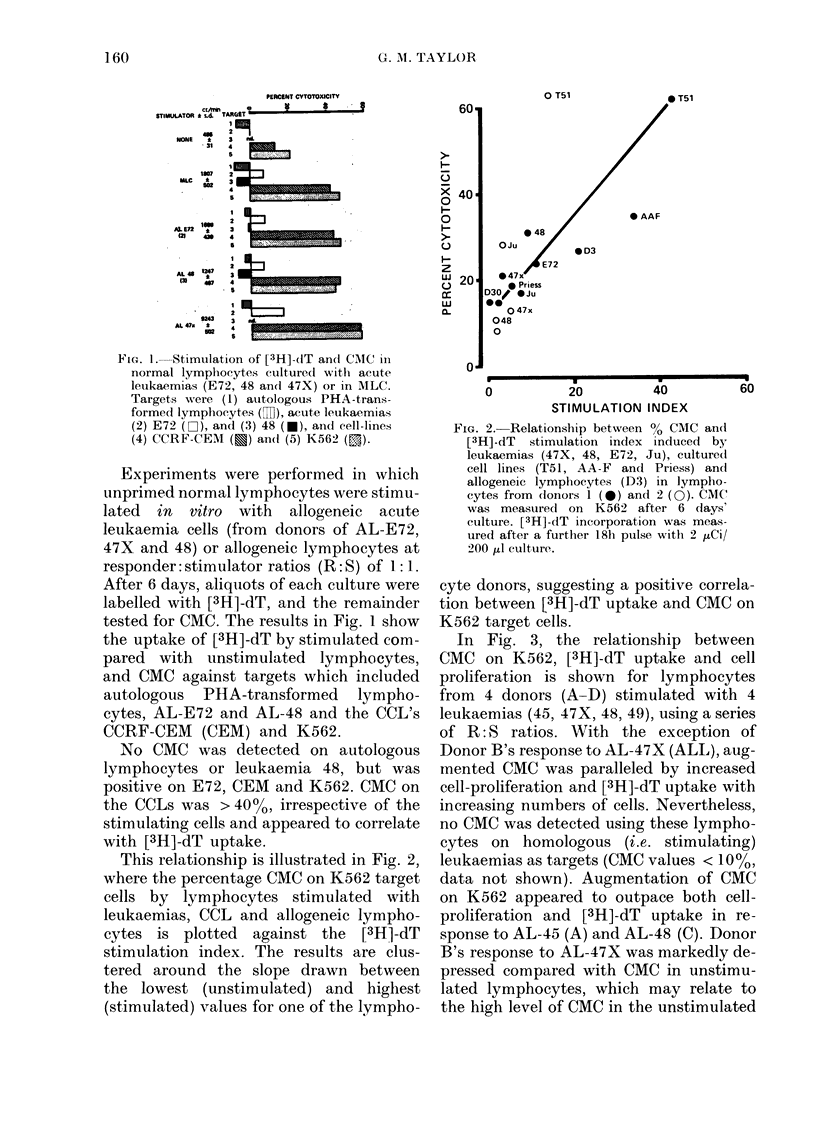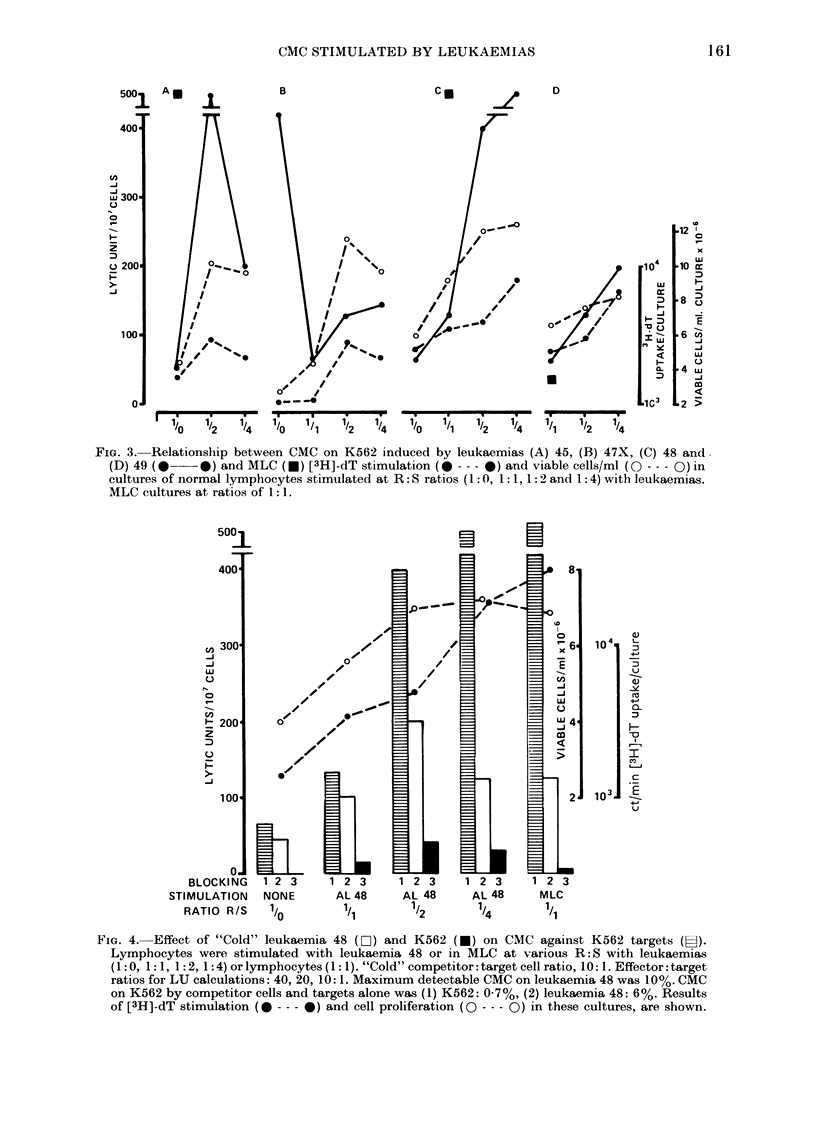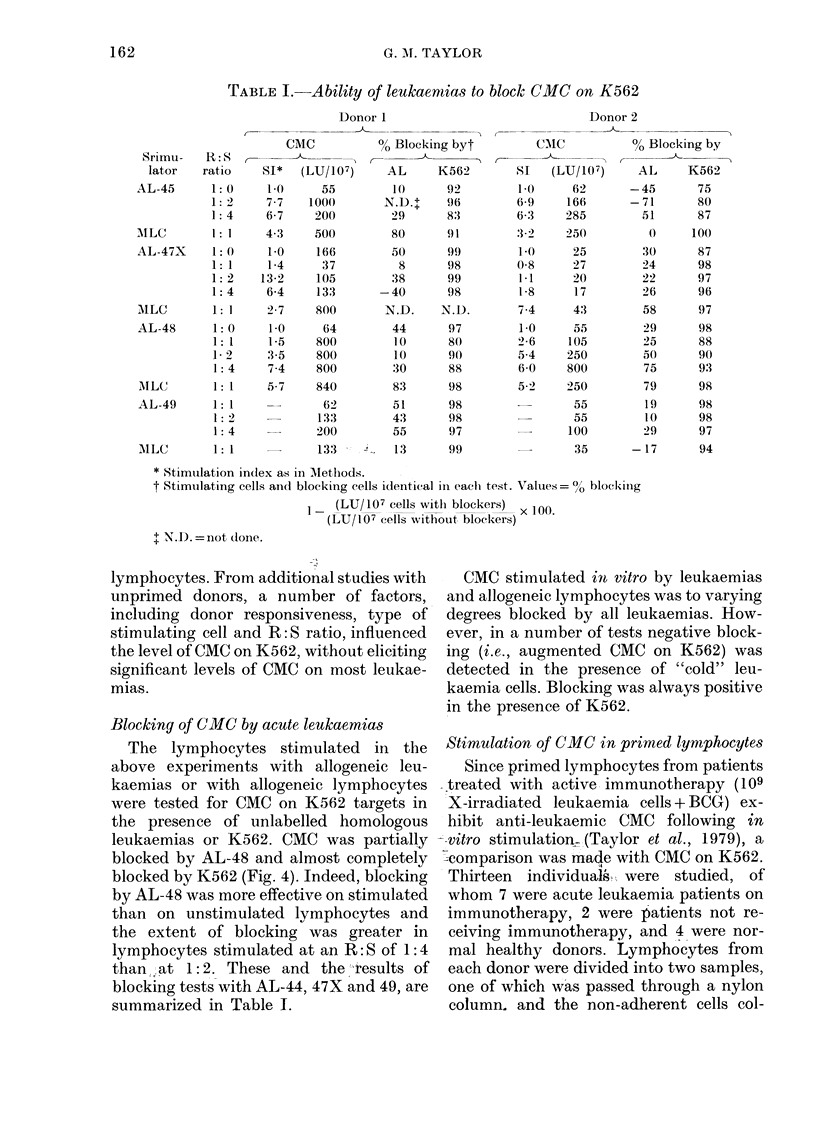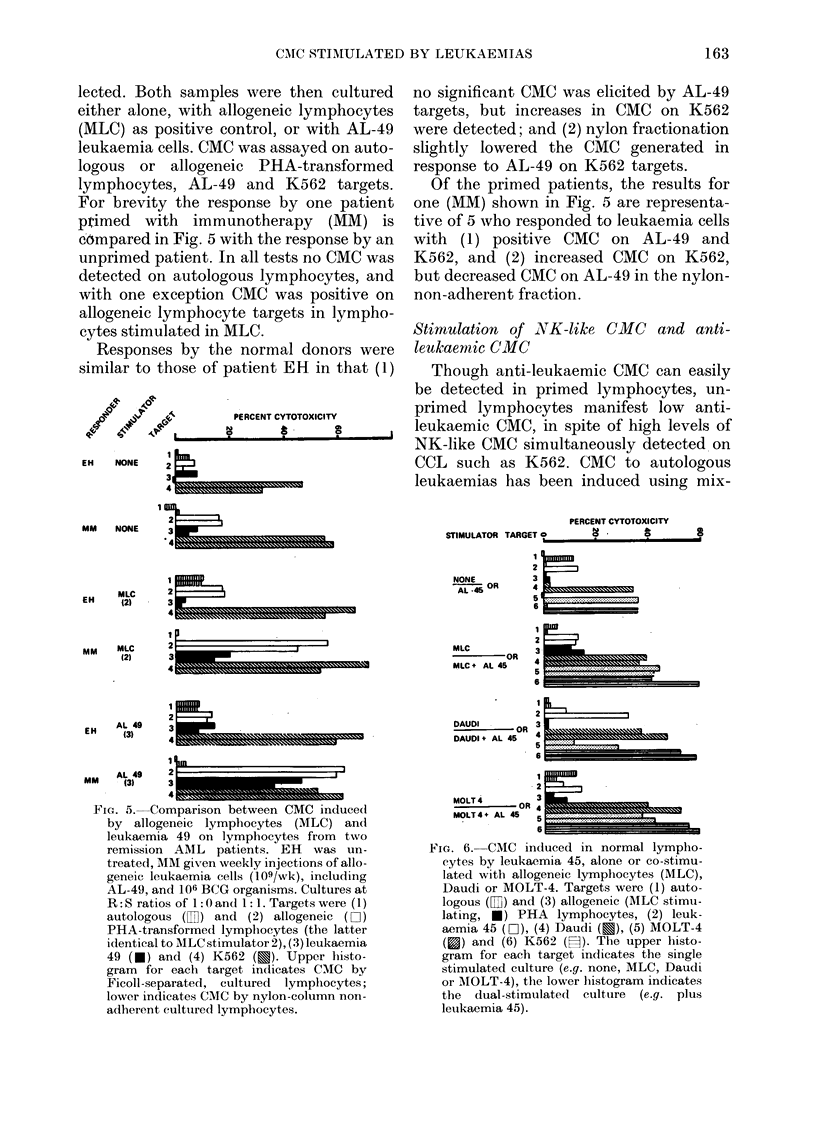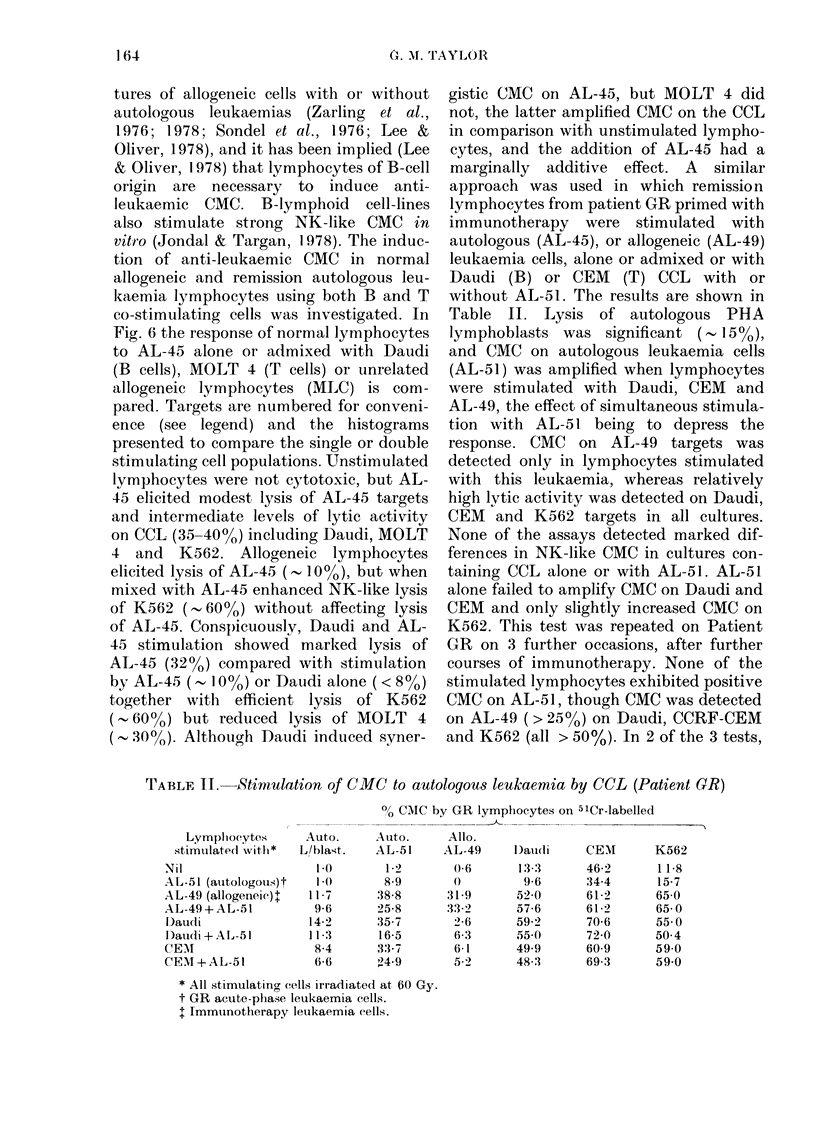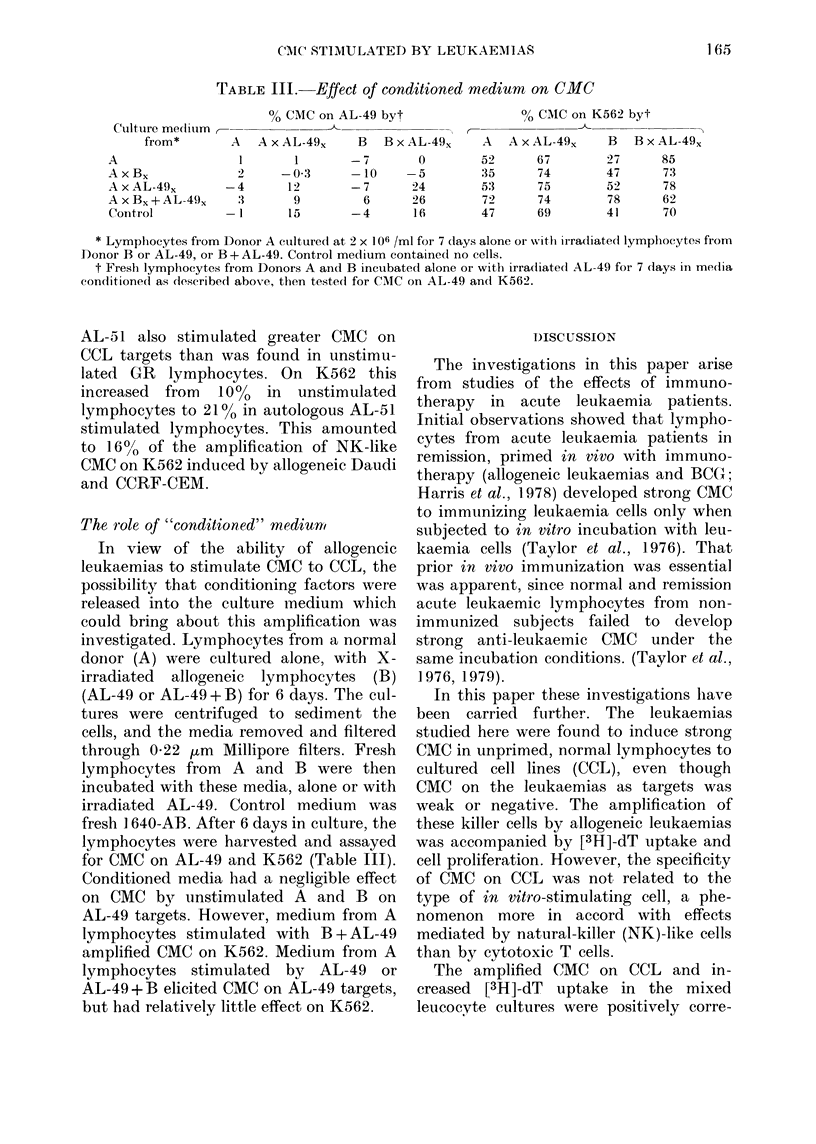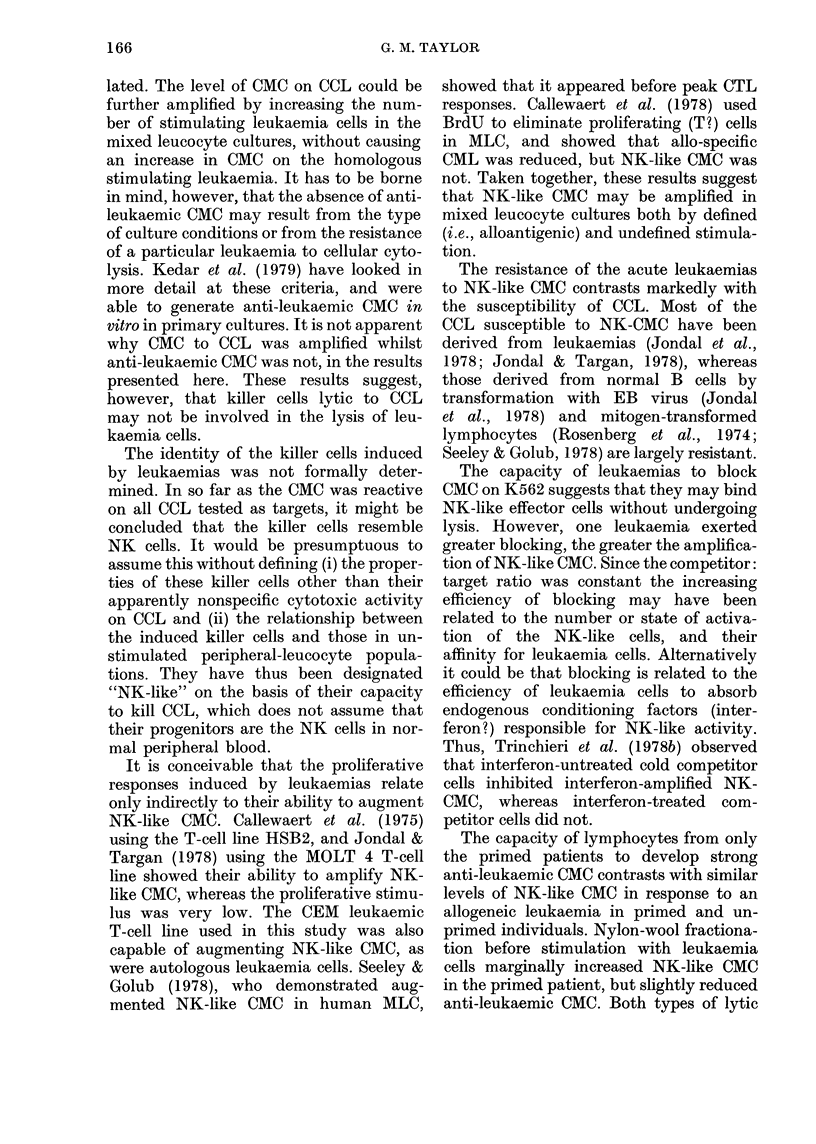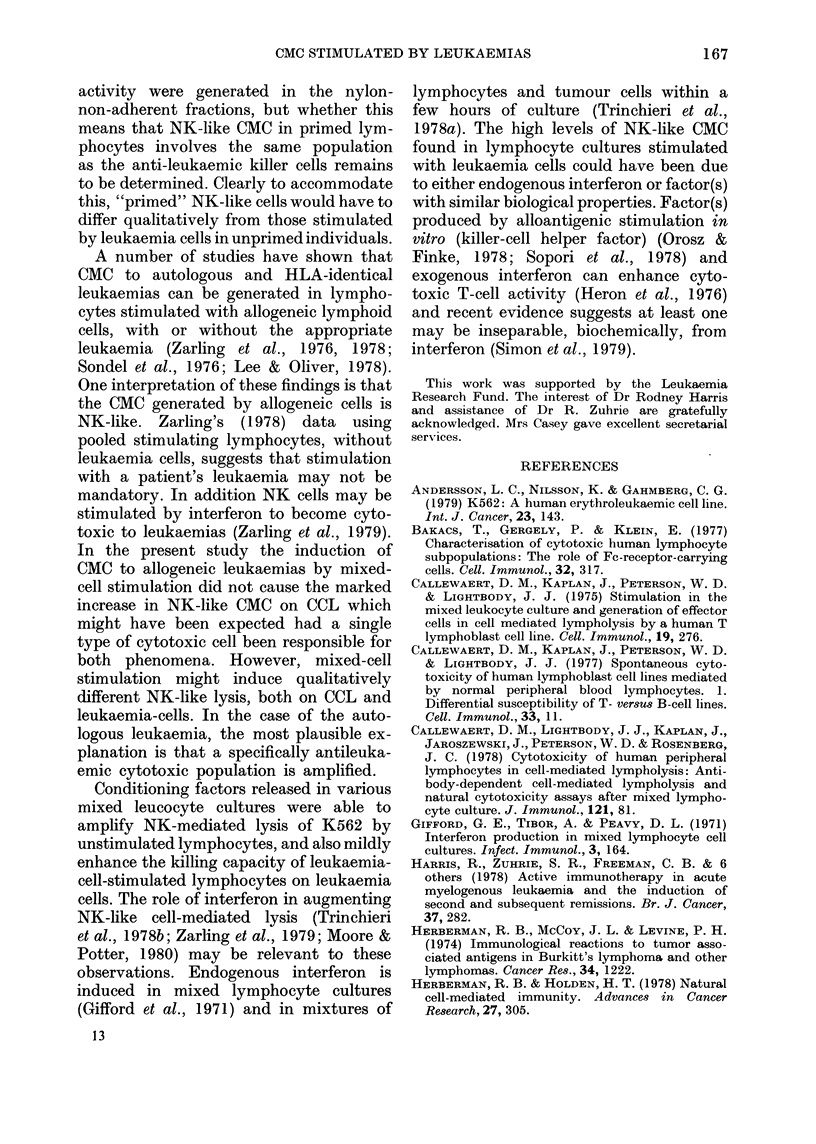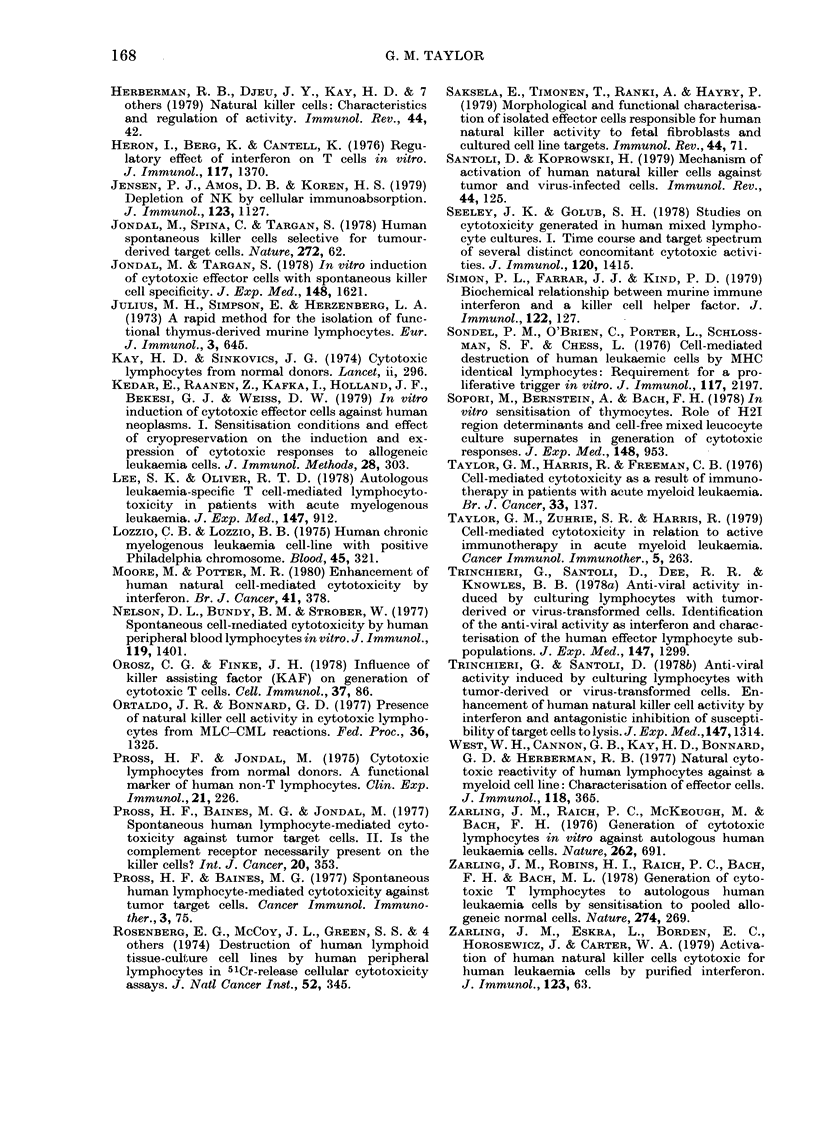# In vitro stimulation of cell-mediated cytotoxicity by acute leukaemias.

**DOI:** 10.1038/bjc.1981.24

**Published:** 1981-02

**Authors:** G. M. Taylor

## Abstract

Acute leukaemias stimulated proliferative and cell-mediated cytotoxic (CMC) responses in vitro in normal (unprimed) lymphocytes. Proliferation was detected by increases in viable cell counts and [3H]dT incorporation in mixed lymphocyte-leukaemia-cell cultures. CMC detected on cultured cell-line targets (CCL) including K562 was generally much stronger than on fresh leukaemia cells, and correlated with stimulation of [3H]dT uptake in the responding lymphocytes. Leukaemias which were resistant as targets to CMC were able competitively to inhibit CMC on K562, though not as efficiently as blocking by K562 itself. With one leukaemia, blocking of CMC increased as the level of CMC on K562 was amplified by greater numbers of stimulating cells in the sensitization phase. This suggests that in certain cases blocking of effector cells by acute-leukaemia cells may depend upon the state of activation of the effector cells. Lymphocytes from a leukaemia patient in remission, treated with allogeneic leukaemia-cell immunotherapy and stimulated in vitro with immunizing leukaemia cells, developed strong anti-leukaemic CMC. A non-immunized patient's lymphocytes did not respond in this way, despite comparable levels of CMC on K562 in both patients. Dual stimulation of unprimed normal lymphocytes and remission lymphocytes with allogeneic or autologous leukaemias and various cell lines, amplified anti-leukaemic CMC, but did not markedly alter CMC or CCL. These data do not formally exclude the mediation of in vitro-stimulated anti-leukaemic CMC by NK-like cells, but suggest that such effector cells differ qualitatively from NK-like cells detected in the absence of anti-leukaemic CMC.


Br. J. Cancer (1981) 43, 157

### *IN VITRO* STIMULATION OF CELL-MEDIATED CYTOTOXICITY BY ACUTE LEUKAEMIAS

G. M. TAYLOR

*From the University of Manchester, Department of Medical Genetics, St Mary's Hospital, Hathersage Road, Manchester M13 0JH, U.K.*

Received 8 September 1980 Accepted 23 October 1980

**Summary.—Acute leukaemias stimulated proliferative and cell-mediated cytotoxic (CMC) responses *in vitro* in normal (unprimed) lymphocytes. Proliferation was detected by increases in viable cell counts and [$^3$H]dT incorporation in mixed lymphocyte–leukaemia-cell cultures. CMC detected on cultured cell-line targets (CCL) including K562 was generally much stronger than on fresh leukaemia cells, and correlated with stimulation of [$^3$H]dT uptake in the responding lymphocytes. Leukaemias which were resistant as targets to CMC were able competitively to inhibit CMC on K562, though not as efficiently as blocking by K562 itself. With one leukaemia, blocking of CMC increased as the level of CMC on K562 was amplified by greater numbers of stimulating cells in the sensitization phase. This suggests that in certain cases blocking of effector cells by acute-leukaemia cells may depend upon the state of activation of the effector cells. Lymphocytes from a leukaemia patient in remission, treated with allogeneic leukaemia-cell immunotherapy and stimulated *in vitro* with immunizing leukaemia cells, developed strong anti-leukaemic CMC. A non-immunized patient's lymphocytes did not respond in this way, despite comparable levels of CMC on K562 in both patients. Dual stimulation of unprimed normal lymphocytes and remission lymphocytes with allogeneic or autologous leukaemias and various cell lines, amplified anti-leukaemic CMC, but did not markedly alter CMC on CCL. These data do not formally exclude the mediation of *in vitro*-stimulated anti-leukaemic CMC by NK-like cells, but suggest that such effector cells differ qualitatively from NK-like cells detected in the absence of anti-leukaemic CMC.**

SINCE THE FIRST EVIDENCE that normal human peripheral-blood leucocytes could manifest direct, spontaneous cell-mediated cytotoxicity (CMC) on $^{51}$Cr-labelled target cells (Kay & Sinkovics, 1974; Rosenberg *et al.*, 1974) evidence has accumulated from a large number of studies (reviewed by Pross & Baines, 1977; Herberman & Holden, 1978) which point to the existence of a distinct population of mononuclear cells with this property. These are now generally referred to as natural killer (NK) cells (Herberman & Holden, 1978). Although the relationship of NK cells to T and B lymphocytes and non-lymphoid mononuclear cells has been the subject of intense investigation (reviewed by Herberman *et al.*, 1979; Saksela *et al.*, 1979; Santoli & Koprowski, 1979) there is general agreement that human NK cells do not express cell-, surface immunoglobulin, or C3 receptors, but do have Fc receptors (Pross & Jondal 1975; Pross *et al.*, 1977; Nelson *et al.*, West *et al.*, 1977; Bakacs *et al.*, 1977) and are non-adherent to plastic surfaces and nylon fibres (Santoli & Koprowski, 1979; Herberman *et al.*, 1979).

NK cells can be detected by their capacity to lyse cultured cell-lines (CCL)

of malignant origin, and speculation has thus focused upon the role that NK cells might play in the prevention of tumour-cell growth *in vivo* (Pross & Baines, 1977; Herberman & Holden, 1978; Santoli & Koprowski, 1979). However, it has yet to be shown conclusively that NK cells are responsible for the lysis of fresh human leukaemia cells, even though spontaneous CMC against autologous acute leukaemias has been reported (Herberman *et al.*, 1974). In this respect procedures for the specific purification of NK cells, such as those based upon absorption on target-cell monolayers (Jensen *et al.*, 1979) are likely to be useful.

Stimulation of human lymphocytes in mixed leucocyte cultures by a variety of cell types leads to augmented CMC on cultured cell lines (Callewaert *et al.*, 1975, 1977; Jondal & Targan, 1978; Ortaldo & Bonnard, 1977). This CMC can be distinguished in its kinetic characteristics from allospecific T-cell cytotoxicity and may be mediated by NK or activated NK cells (Seeley & Golub, 1978). Lymphocytes from acute leukaemia patients in remission, stimulated *in vitro* with allogeneic acute leukaemia cells, develop CMC to fresh leukaemias only when patients have been immunized *in vivo* with leukaemia-cell immunotherapy (Taylor *et al.*, 1979). The purpose of this study was to assess the capacity of different leukaemias to amplify *in vitro* or block CMC in normal and primed lymphocytes on leukaemia and CCL targets as a means of determining the relationship between anti-leukaemic CMC and CMC on cell lines.

### MATERIALS AND METHODS

### *Preparation of lymphocytes*

Blood donors included normal individuals and acute leukaemia patients in remission. Remission induction and immunotherapy of the leukaemia patients have been described (Taylor *et al.*, 1979). Defibrinated blood was diluted 1:1 with Hanks' Balanced Salt Solution (HBSS) and separated on lymphocyte-separation medium (LSM, sp. gr. 1077, Flow Laboratories, Irvine, Scotland). Lymphocytes obtained at the LSM–plasma interface were diluted with Eagle's medium + foetal calf serum (MEM–FCS) and sedimented by centrifugation. The lymphocytes were re-suspended in MEM–FCS, washed and re-suspended in RPMI-1640 containing 10% heat-inactivated AB serum (1640–AB).

### *Cultured cell lines (CCL)*

The CCL used in this study were K562, an erythroleukaemia (Lozzio & Lozzio, 1975; Andersson *et al.*, 1979), CCRF–CEM and MOLT 4 (T-cell leukaemias), Daudi (Burkitt's lymphoma) AA-F, T51 and PRIESS (B lymphoblastoid cells). They were cultured in 500ml glass bottles in ~100 ml of 1640–FCS (10%) and antibiotics at 37°C in a 95% air/5% $CO_2$ gas mixture. Media were changed twice weekly, and only actively growing cells with >95% viability were used as targets.

### *Leukaemia cells*

The procurement of leukaemia cells, their separation from peripheral blood, freezing and storage have been described elsewhere (Taylor *et al.*, 1979). The cells are denoted by their laboratory codes and are of the following diagnostic types: AL–45 (AML), AL–47X (ALL), AL–48 (AMoL), AL–49 (AMML), AL–51 (AML), AL–E72 (AMML).

Abbreviations: AML = acute myeloblastic leukaemia; AMoL = acute monoblastic leukaemia; ALL = acute lymphoblastic leukaemia; AMML = acute myelomonocytic leukaemia; AL = acute leukaemia.

### *Nylon-column separation*

Lymphocytes were separated into nylon-adherent and non-adherent cells by passage through nylon columns, according to the method of Julius *et al.* (1973). Briefly, washed nylon from a Fenwal Leucopak (Travenol Laboratories) was packed to the 1ml mark of a 5ml syringe and incubated with 5 ml of 1640–HEPES for 30 min at 37°C. One ml of lymphocyte suspension (2–4 × $10^7$ cells) was added and the column incubated for 30 min at 37°C. The non-adhering lymphocytes were washed through with warm 1640–HEPES–FCS and collected in a final volume of 10 ml. They were sedimented by centrifugation and resuspended in 1640–AB for culture.

*Mixed leucocyte cultures*

Unfiltered or nylon-non-adherent lymphocytes were adjusted to $2 \times 10^6$/ml for use as responding cells. Stimulating cells were CCL, leukaemia cells or allogeneic lymphocytes, treated either with Mitomycin C (MC, Sigma Chemical Co., Kingston-upon-Thames) at a concentration of 50–100 $\mu$g/$10^7$ cells for 30 min at 37°C, or with 60 Gy from a $^{137}$Cs source. Stimulating cells were washed 3× after MC treatment, but not after irradiation, and adjusted to $2 \times 10^6$/ml. Equal volumes (2 ml) of responding and stimulating cells were mixed in round-bottomed plastic tissue-culture tubes (Sterilin, Teddington). The tubes were gassed with 95% air/5% $CO_2$, transferred to a humidified Gas-Pak 150 jar (Beckton–Dickinson, Wembley) and incubated at 37°C for 6 days (day of culture = Day 0). Replicate cultures were then harvested and pooled, washed once in 1640–HEPES–FCS and adjusted to cell concentrations appropriate for CMC assays.

*Cell-mediated cytotoxicity*

*Target cells.*—These included PHA-transformed lymphocytes, CCL and leukaemia cells. Normal lymphocytes ($10^6$/ml) were transformed by incubation for 3 days with PHA (Difco PHA-P, 10 $\mu$l of a 1/100 diluted stock solution/ml culture). Leukaemia cells and CCL were prepared as described previously (Taylor *et al.*, 1979). All target cells were labelled with 200 $\mu$Ci $^{51}$Cr (CJS 1P, sp. act. 1 mCi/mol., Radiochemical Centre, Amersham) for 1 h at 20°C. The targets were then washed once by centrifugation in 1640–HEPES–FCS, the sedimented cells resuspended in 10 ml of the same medium and reincubated for a further hour at 20°C. The cells were then washed twice, and the concentration adjusted to $5 \times 10^4$/ml or $10^5$/ml for CMC assays.

*CMC assay.*—One hundred $\mu$l of effector cells were mixed with 100 $\mu$l of the appropriate targets in microplates with 96 round-bottomed wells (M24ART, Sterilin). Each effector: target combination was tested in triplicate, at a ratio of 50:1 or, for the calculation of lytic units (LU) at ratios of 40, 20 and 10:1.

Blocking of CMC by "cold" targets was performed by adding 100 $\mu$l of cultured lymphocytes and 50 $\mu$l of unlabelled target cells ($2 \times 10^5$/ml) to each microplate well. The plates were spun at 60 *g*, incubated for 30 min at 37°C and 50 $\mu$l of $^{51}$Cr-labelled K562 targets ($2 \times 10^4$/ml) then added, giving a blocker: target-cell ratio of 10:1.

CMC assays were incubated at 37°C for 6 h in a humidified Gas-Pak 150 jar, then spun at 60 *g* for 5 min, and supernatants harvested with a SKATRON/Titertek supernatant harvester (Flow Laboratories). $^{51}$Cr in the supernatants was detected on a Wallac DECEM GTL 300–500 gamma scintillation counter.

Percentage cytotoxicity (% CMC) was calculated from: $E - S/T - S \times 100$, where E = experimental $^{51}$Cr release in the presence of lymphocytes, S = spontaneous $^{51}$Cr released by target cells alone, and T = total $^{51}$Cr released by adding 2% Triton ×100 and freeze-thawing ×3.

Lytic units (LU) were calculated from slopes of % CMC against effector:target (E:T) ratio. One LU was the number of effector cells needed to give 20% cytotoxicity on K562 target cells. Results are expressed as LU/$10^7$ effector cells.

*$^3$H-Thymidine incorporation.*—Mixed cell cultures were resuspended after 6 days and 200 $\mu$l of each culture transferred to each of 4 microplate wells (M24ART). Each well was labelled with 2 $\mu$Ci [$^3$H]-dT (TRK–120 sp. act. 20 mCi/mmol, Radiochemical Centre, Amersham). The microplates were incubated (as above) for 18 h and harvested on to glass-fibre filters with a SKATRON/Titertek cell harvester (Flow Laboratories). The filters were dried overnight at 37°C in polypropylene scintillation vials, then 5 ml of toluene-based scintillation cocktail was added and [$^3$H]-dT uptake counted on a Beckman LS-3155T liquid scintillation counter. The results (mean ct/min ± s.d.) are in some cases expressed as stimulation indices, with ct/min in stimulated lymphocytes divided by that in unstimulated controls.

### RESULTS

*Stimulation of CMC by acute leukaemias*

Previous results showed that both unprimed and *in vivo*-primed remission lymphocytes from leukaemia patients receiving immunotherapy developed CMC to CCL after *in vitro* stimulation with allogeneic leukaemia cells (Taylor *et al.*, 1979). This was investigated in greater detail as follows.

FIG. 1.—Stimulation of [$^3$H]-dT and CMC in normal lymphocytes cultured with acute leukaemias (E72, 48 and 47X) or in MLC. Targets were (1) autologous PHA-transformed lymphocytes (▥), acute leukaemias (2) E72 (□), and (3) 48 (■), and cell-lines (4) CCRF-CEM (▧) and (5) K562 (▨).

FIG. 2.—Relationship between % CMC and [$^3$H]-dT stimulation index induced by leukaemias (47X, 48, E72, Ju), cultured cell lines (T51, AA-F and Priess) and allogeneic lymphocytes (D3) in lymphocytes from donors 1 (●) and 2 (○). CMC was measured on K562 after 6 days' culture. [$^3$H]-dT incorporation was measured after a further 18h pulse with 2 $\mu$Ci/200 $\mu$l culture.

Experiments were performed in which unprimed normal lymphocytes were stimulated *in vitro* with allogeneic acute leukaemia cells (from donors of AL-E72, 47X and 48) or allogeneic lymphocytes at responder:stimulator ratios (R:S) of 1:1. After 6 days, aliquots of each culture were labelled with [$^3$H]-dT, and the remainder tested for CMC. The results in Fig. 1 show the uptake of [$^3$H]-dT by stimulated compared with unstimulated lymphocytes, and CMC against targets which included autologous PHA-transformed lymphocytes, AL-E72 and AL-48 and the CCL's CCRF-CEM (CEM) and K562.

No CMC was detected on autologous lymphocytes or leukaemia 48, but was positive on E72, CEM and K562. CMC on the CCLs was $>40\%$, irrespective of the stimulating cells and appeared to correlate with [$^3$H]-dT uptake.

This relationship is illustrated in Fig. 2, where the percentage CMC on K562 target cells by lymphocytes stimulated with leukaemias, CCL and allogeneic lymphocytes is plotted against the [$^3$H]-dT stimulation index. The results are clustered around the slope drawn between the lowest (unstimulated) and highest (stimulated) values for one of the lymphocyte donors, suggesting a positive correlation between [$^3$H]-dT uptake and CMC on K562 target cells.

In Fig. 3, the relationship between CMC on K562, [$^3$H]-dT uptake and cell proliferation is shown for lymphocytes from 4 donors (A–D) stimulated with 4 leukaemias (45, 47X, 48, 49), using a series of R:S ratios. With the exception of Donor B's response to AL-47X (ALL), augmented CMC was paralleled by increased cell-proliferation and [$^3$H]-dT uptake with increasing numbers of cells. Nevertheless, no CMC was detected using these lymphocytes on homologous (*i.e.* stimulating) leukaemias as targets (CMC values $<10\%$, data not shown). Augmentation of CMC on K562 appeared to outpace both cell-proliferation and [$^3$H]-dT uptake in response to AL-45 (A) and AL-48 (C). Donor B's response to AL-47X was markedly depressed compared with CMC in unstimulated lymphocytes, which may relate to the high level of CMC in the unstimulated

FIG. 3.—Relationship between CMC on K562 induced by leukaemias (A) 45, (B) 47X, (C) 48 and (D) 49 (●——●) and MLC (■) [$^3$H]-dT stimulation (● - - - ●) and viable cells/ml (○ - - - ○) in cultures of normal lymphocytes stimulated at R:S ratios (1:0, 1:1, 1:2 and 1:4) with leukaemias. MLC cultures at ratios of 1:1.

FIG. 4.—Effect of "Cold" leukaemia 48 (□) and K562 (■) on CMC against K562 targets (▤). Lymphocytes were stimulated with leukaemia 48 or in MLC at various R:S with leukaemias (1:0, 1:1, 1:2, 1:4) or lymphocytes (1:1). "Cold" competitor:target cell ratio, 10:1. Effector:target ratios for LU calculations: 40, 20, 10:1. Maximum detectable CMC on leukaemia 48 was 10%. CMC on K562 by competitor cells and targets alone was (1) K562: 0·7%, (2) leukaemia 48: 6%. Results of [$^3$H]-dT stimulation (● - - - ●) and cell proliferation (○ - - - ○) in these cultures, are shown.

TABLE I.—*Ability of leukaemias to block CMC on K*562

| Srimulator | R:S ratio | Donor 1 CMC SI* | Donor 1 CMC (LU/$10^7$) | Donor 1 % Blocking by† AL | Donor 1 % Blocking by† K562 | Donor 2 CMC SI | Donor 2 CMC (LU/$10^7$) | Donor 2 % Blocking by AL | Donor 2 % Blocking by K562 |
|---|---|---|---|---|---|---|---|---|---|
| AL-45 | 1:0 | 1·0 | 55 | 10 | 92 | 1·0 | 62 | −45 | 75 |
| | 1:2 | 7·7 | 1000 | N.D.‡ | 96 | 6·9 | 166 | −71 | 80 |
| | 1:4 | 6·7 | 200 | 29 | 83 | 6·3 | 285 | 51 | 87 |
| MLC | 1:1 | 4·3 | 500 | 80 | 91 | 3·2 | 250 | 0 | 100 |
| AL-47X | 1:0 | 1·0 | 166 | 50 | 99 | 1·0 | 25 | 30 | 87 |
| | 1:1 | 1·4 | 37 | 8 | 98 | 0·8 | 27 | 24 | 98 |
| | 1:2 | 13·2 | 105 | 38 | 99 | 1·1 | 20 | 22 | 97 |
| | 1:4 | 6·4 | 133 | −40 | 98 | 1·8 | 17 | 26 | 96 |
| MLC | 1:1 | 2·7 | 800 | N.D. | N.D. | 7·4 | 43 | 58 | 97 |
| AL-48 | 1:0 | 1·0 | 64 | 44 | 97 | 1·0 | 55 | 29 | 98 |
| | 1:1 | 1·5 | 800 | 10 | 80 | 2·6 | 105 | 25 | 88 |
| | 1:2 | 3·5 | 800 | 10 | 90 | 5·4 | 250 | 50 | 90 |
| | 1:4 | 7·4 | 800 | 30 | 88 | 6·0 | 800 | 75 | 93 |
| MLC | 1:1 | 5·7 | 840 | 83 | 98 | 5·2 | 250 | 79 | 98 |
| AL-49 | 1:1 | — | 62 | 51 | 98 | — | 55 | 19 | 98 |
| | 1:2 | — | 133 | 43 | 98 | — | 55 | 10 | 98 |
| | 1:4 | — | 200 | 55 | 97 | — | 100 | 29 | 97 |
| MLC | 1:1 | — | 133 | 13 | 99 | — | 35 | −17 | 94 |

* Stimulation index as in Methods.

† Stimulating cells and blocking cells identical in each test. Values = % blocking

$$1-\frac{(\text{LU}/10^7 \text{ cells with blockers})}{(\text{LU}/10^7 \text{ cells without blockers})}\times 100.$$

‡ N.D. = not done.

lymphocytes. From additional studies with unprimed donors, a number of factors, including donor responsiveness, type of stimulating cell and R:S ratio, influenced the level of CMC on K562, without eliciting significant levels of CMC on most leukaemias.

### *Blocking of CMC by acute leukaemias*

The lymphocytes stimulated in the above experiments with allogeneic leukaemias or with allogeneic lymphocytes were tested for CMC on K562 targets in the presence of unlabelled homologous leukaemias or K562. CMC was partially blocked by AL-48 and almost completely blocked by K562 (Fig. 4). Indeed, blocking by AL-48 was more effective on stimulated than on unstimulated lymphocytes and the extent of blocking was greater in lymphocytes stimulated at an R:S of 1:4 than at 1:2. These and the results of blocking tests with AL-44, 47X and 49, are summarized in Table I.

CMC stimulated *in vitro* by leukaemias and allogeneic lymphocytes was to varying degrees blocked by all leukaemias. However, in a number of tests negative blocking (*i.e.*, augmented CMC on K562) was detected in the presence of "cold" leukaemia cells. Blocking was always positive in the presence of K562.

### *Stimulation of CMC in primed lymphocytes*

Since primed lymphocytes from patients treated with active immunotherapy ($10^9$ X-irradiated leukaemia cells + BCG) exhibit anti-leukaemic CMC following *in vitro* stimulation (Taylor *et al.*, 1979), a comparison was made with CMC on K562. Thirteen individuals were studied, of whom 7 were acute leukaemia patients on immunotherapy, 2 were patients not receiving immunotherapy, and 4 were normal healthy donors. Lymphocytes from each donor were divided into two samples, one of which was passed through a nylon column. and the non-adherent cells collected. Both samples were then cultured either alone, with allogeneic lymphocytes (MLC) as positive control, or with AL-49 leukaemia cells. CMC was assayed on autologous or allogeneic PHA-transformed lymphocytes, AL-49 and K562 targets. For brevity the response by one patient primed with immunotherapy (MM) is compared in Fig. 5 with the response by an unprimed patient. In all tests no CMC was detected on autologous lymphocytes, and with one exception CMC was positive on allogeneic lymphocyte targets in lymphocytes stimulated in MLC.

Responses by the normal donors were similar to those of patient EH in that (1) no significant CMC was elicited by AL-49 targets, but increases in CMC on K562 were detected; and (2) nylon fractionation slightly lowered the CMC generated in response to AL-49 on K562 targets.

Of the primed patients, the results for one (MM) shown in Fig. 5 are representative of 5 who responded to leukaemia cells with (1) positive CMC on AL-49 and K562, and (2) increased CMC on K562, but decreased CMC on AL-49 in the nylon-non-adherent fraction.

FIG. 5.—Comparison between CMC induced by allogeneic lymphocytes (MLC) and leukaemia 49 on lymphocytes from two remission AML patients. EH was untreated, MM given weekly injections of allogeneic leukaemia cells ($10^9$/wk), including AL-49, and $10^6$ BCG organisms. Cultures at R:S ratios of 1:0 and 1:1. Targets were (1) autologous (▥) and (2) allogeneic (□) PHA-transformed lymphocytes (the latter identical to MLC stimulator 2), (3) leukaemia 49 (■) and (4) K562 (▧). Upper histogram for each target indicates CMC by Ficoll-separated, cultured lymphocytes; lower indicates CMC by nylon-column non-adherent cultured lymphocytes.

*Stimulation of NK-like CMC and anti-leukaemic CMC*

Though anti-leukaemic CMC can easily be detected in primed lymphocytes, unprimed lymphocytes manifest low anti-leukaemic CMC, in spite of high levels of NK-like CMC simultaneously detected on CCL such as K562. CMC to autologous leukaemias has been induced using mix-

FIG. 6.—CMC induced in normal lymphocytes by leukaemia 45, alone or co-stimulated with allogeneic lymphocytes (MLC), Daudi or MOLT-4. Targets were (1) autologous (▥) and (3) allogeneic (MLC stimulating, ■) PHA lymphocytes, (2) leukaemia 45 (□), (4) Daudi (▧), (5) MOLT-4 (▨) and (6) K562 (▤). The upper histogram for each target indicates the single stimulated culture (*e.g.* none, MLC, Daudi or MOLT-4), the lower histogram indicates the dual-stimulated culture (*e.g.* plus leukaemia 45).

tures of allogeneic cells with or without autologous leukaemias (Zarling *et al.*, 1976; 1978; Sondel *et al.*, 1976; Lee & Oliver, 1978), and it has been implied (Lee & Oliver, 1978) that lymphocytes of B-cell origin are necessary to induce anti-leukaemic CMC. B-lymphoid cell-lines also stimulate strong NK-like CMC *in vitro* (Jondal & Targan, 1978). The induction of anti-leukaemic CMC in normal allogeneic and remission autologous leukaemia lymphocytes using both B and T co-stimulating cells was investigated. In Fig. 6 the response of normal lymphocytes to AL-45 alone or admixed with Daudi (B cells), MOLT 4 (T cells) or unrelated allogeneic lymphocytes (MLC) is compared. Targets are numbered for convenience (see legend) and the histograms presented to compare the single or double stimulating cell populations. Unstimulated lymphocytes were not cytotoxic, but AL-45 elicited modest lysis of AL-45 targets and intermediate levels of lytic activity on CCL (35–40%) including Daudi, MOLT 4 and K562. Allogeneic lymphocytes elicited lysis of AL-45 (~10%), but when mixed with AL-45 enhanced NK-like lysis of K562 (~60%) without affecting lysis of AL-45. Conspicuously, Daudi and AL-45 stimulation showed marked lysis of AL-45 (32%) compared with stimulation by AL-45 (~10%) or Daudi alone (<8%) together with efficient lysis of K562 (~60%) but reduced lysis of MOLT 4 (~30%). Although Daudi induced synergistic CMC on AL-45, but MOLT 4 did not, the latter amplified CMC on the CCL in comparison with unstimulated lymphocytes, and the addition of AL-45 had a marginally additive effect. A similar approach was used in which remission lymphocytes from patient GR primed with immunotherapy were stimulated with autologous (AL-45), or allogeneic (AL-49) leukaemia cells, alone or admixed or with Daudi (B) or CEM (T) CCL with or without AL-51. The results are shown in Table II. Lysis of autologous PHA lymphoblasts was significant (~15%), and CMC on autologous leukaemia cells (AL-51) was amplified when lymphocytes were stimulated with Daudi, CEM and AL-49, the effect of simultaneous stimulation with AL-51 being to depress the response. CMC on AL-49 targets was detected only in lymphocytes stimulated with this leukaemia, whereas relatively high lytic activity was detected on Daudi, CEM and K562 targets in all cultures. None of the assays detected marked differences in NK-like CMC in cultures containing CCL alone or with AL-51. AL-51 alone failed to amplify CMC on Daudi and CEM and only slightly increased CMC on K562. This test was repeated on Patient GR on 3 further occasions, after further courses of immunotherapy. None of the stimulated lymphocytes exhibited positive CMC on AL-51, though CMC was detected on AL-49 (>25%) on Daudi, CCRF-CEM and K562 (all >50%). In 2 of the 3 tests,

TABLE II.—*Stimulation of CMC to autologous leukaemia by CCL (Patient GR)*

| Lymphocytes stimulated with* | % CMC by GR lymphocytes on $^{51}$Cr-labelled | | | | | |
|---|---|---|---|---|---|---|
| | Auto. L/blast. | Auto. AL-51 | Allo. AL-49 | Daudi | CEM | K562 |
| Nil | 1·0 | 1·2 | 0·6 | 13·3 | 46·2 | 11·8 |
| AL-51 (autologous)† | 1·0 | 8·9 | 0 | 9·6 | 34·4 | 15·7 |
| AL-49 (allogeneic)‡ | 11·7 | 38·8 | 31·9 | 52·0 | 61·2 | 65·0 |
| AL-49+AL-51 | 9·6 | 25·8 | 33·2 | 57·6 | 61·2 | 65·0 |
| Daudi | 14·2 | 35·7 | 2·6 | 59·2 | 70·6 | 55·0 |
| Daudi+AL-51 | 11·3 | 16·5 | 6·3 | 55·0 | 72·0 | 50·4 |
| CEM | 8·4 | 33·7 | 6·1 | 49·9 | 60·9 | 59·0 |
| CEM+AL-51 | 6·6 | 24·9 | 5·2 | 48·3 | 69·3 | 59·0 |

* All stimulating cells irradiated at 60 Gy.
† GR acute-phase leukaemia cells.
‡ Immunotherapy leukaemia cells.

TABLE III.—*Effect of conditioned medium on CMC*

| Culture medium from* | % CMC on AL-49 by† | | | | % CMC on K562 by† | | | |
|---|---|---|---|---|---|---|---|---|
| | A | A x AL-$49_x$ | B | B x AL-$49_x$ | A | A x AL-$49_x$ | B | B x AL-$49_x$ |
| A | 1 | 1 | −7 | 0 | 52 | 67 | 27 | 85 |
| A x $B_x$ | 2 | −0·3 | −10 | −5 | 35 | 74 | 47 | 73 |
| A x AL-$49_x$ | −4 | 12 | −7 | 24 | 53 | 75 | 52 | 78 |
| A x $B_x$ + AL-$49_x$ | 3 | 9 | 6 | 26 | 72 | 74 | 78 | 62 |
| Control | −1 | 15 | −4 | 16 | 47 | 69 | 41 | 70 |

* Lymphocytes from Donor A cultured at $2 \times 10^6$ /ml for 7 days alone or with irradiated lymphocytes from Donor B or AL-49, or B + AL-49. Control medium contained no cells.

† Fresh lymphocytes from Donors A and B incubated alone or with irradiated AL-49 for 7 days in media conditioned as described above, then tested for CMC on AL-49 and K562.

AL-51 also stimulated greater CMC on CCL targets than was found in unstimulated GR lymphocytes. On K562 this increased from 10% in unstimulated lymphocytes to 21% in autologous AL-51 stimulated lymphocytes. This amounted to 16% of the amplification of NK-like CMC on K562 induced by allogeneic Daudi and CCRF-CEM.

### *The role of "conditioned" medium*

In view of the ability of allogencic leukaemias to stimulate CMC to CCL, the possibility that conditioning factors were released into the culture medium which could bring about this amplification was investigated. Lymphocytes from a normal donor (A) were cultured alone, with X-irradiated allogeneic lymphocytes (B) (AL-49 or AL-49 + B) for 6 days. The cultures were centrifuged to sediment the cells, and the media removed and filtered through 0·22 μm Millipore filters. Fresh lymphocytes from A and B were then incubated with these media, alone or with irradiated AL-49. Control medium was fresh 1640-AB. After 6 days in culture, the lymphocytes were harvested and assayed for CMC on AL-49 and K562 (Table III). Conditioned media had a negligible effect on CMC by unstimulated A and B on AL-49 targets. However, medium from A lymphocytes stimulated with B + AL-49 amplified CMC on K562. Medium from A lymphocytes stimulated by AL-49 or AL-49 + B elicited CMC on AL-49 targets, but had relatively little effect on K562.

### DISCUSSION

The investigations in this paper arise from studies of the effects of immunotherapy in acute leukaemia patients. Initial observations showed that lymphocytes from acute leukaemia patients in remission, primed *in vivo* with immunotherapy (allogeneic leukaemias and BCG; Harris *et al.*, 1978) developed strong CMC to immunizing leukaemia cells only when subjected to *in vitro* incubation with leukaemia cells (Taylor *et al.*, 1976). That prior *in vivo* immunization was essential was apparent, since normal and remission acute leukaemic lymphocytes from non-immunized subjects failed to develop strong anti-leukaemic CMC under the same incubation conditions. (Taylor *et al.*, 1976, 1979).

In this paper these investigations have been carried further. The leukaemias studied here were found to induce strong CMC in unprimed, normal lymphocytes to cultured cell lines (CCL), even though CMC on the leukaemias as targets was weak or negative. The amplification of these killer cells by allogeneic leukaemias was accompanied by [$^3$H]-dT uptake and cell proliferation. However, the specificity of CMC on CCL was not related to the type of *in vitro*-stimulating cell, a phenomenon more in accord with effects mediated by natural-killer (NK)-like cells than by cytotoxic T cells.

The amplified CMC on CCL and increased [$^3$H]-dT uptake in the mixed leucocyte cultures were positively correlated. The level of CMC on CCL could be further amplified by increasing the number of stimulating leukaemia cells in the mixed leucocyte cultures, without causing an increase in CMC on the homologous stimulating leukaemia. It has to be borne in mind, however, that the absence of anti-leukaemic CMC may result from the type of culture conditions or from the resistance of a particular leukaemia to cellular cytolysis. Kedar *et al.* (1979) have looked in more detail at these criteria, and were able to generate anti-leukaemic CMC *in vitro* in primary cultures. It is not apparent why CMC to CCL was amplified whilst anti-leukaemic CMC was not, in the results presented here. These results suggest, however, that killer cells lytic to CCL may not be involved in the lysis of leukaemia cells.

The identity of the killer cells induced by leukaemias was not formally determined. In so far as the CMC was reactive on all CCL tested as targets, it might be concluded that the killer cells resemble NK cells. It would be presumptuous to assume this without defining (i) the properties of these killer cells other than their apparently nonspecific cytotoxic activity on CCL and (ii) the relationship between the induced killer cells and those in unstimulated peripheral-leucocyte populations. They have thus been designated "NK-like" on the basis of their capacity to kill CCL, which does not assume that their progenitors are the NK cells in normal peripheral blood.

It is conceivable that the proliferative responses induced by leukaemias relate only indirectly to their ability to augment NK-like CMC. Callewaert *et al.* (1975) using the T-cell line HSB2, and Jondal & Targan (1978) using the MOLT 4 T-cell line showed their ability to amplify NK-like CMC, whereas the proliferative stimulus was very low. The CEM leukaemic T-cell line used in this study was also capable of augmenting NK-like CMC, as were autologous leukaemia cells. Seeley & Golub (1978), who demonstrated augmented NK-like CMC in human MLC, showed that it appeared before peak CTL responses. Callewaert *et al.* (1978) used BrdU to eliminate proliferating (T?) cells in MLC, and showed that allo-specific CML was reduced, but NK-like CMC was not. Taken together, these results suggest that NK-like CMC may be amplified in mixed leucocyte cultures both by defined (*i.e.*, alloantigenic) and undefined stimulation.

The resistance of the acute leukaemias to NK-like CMC contrasts markedly with the susceptibility of CCL. Most of the CCL susceptible to NK-CMC have been derived from leukaemias (Jondal *et al.*, 1978; Jondal & Targan, 1978), whereas those derived from normal B cells by transformation with EB virus (Jondal *et al.*, 1978) and mitogen-transformed lymphocytes (Rosenberg *et al.*, 1974; Seeley & Golub, 1978) are largely resistant.

The capacity of leukaemias to block CMC on K562 suggests that they may bind NK-like effector cells without undergoing lysis. However, one leukaemia exerted greater blocking, the greater the amplification of NK-like CMC. Since the competitor: target ratio was constant the increasing efficiency of blocking may have been related to the number or state of activation of the NK-like cells, and their affinity for leukaemia cells. Alternatively it could be that blocking is related to the efficiency of leukaemia cells to absorb endogenous conditioning factors (interferon?) responsible for NK-like activity. Thus, Trinchieri *et al.* (1978*b*) observed that interferon-untreated cold competitor cells inhibited interferon-amplified NK-CMC, whereas interferon-treated competitor cells did not.

The capacity of lymphocytes from only the primed patients to develop strong anti-leukaemic CMC contrasts with similar levels of NK-like CMC in response to an allogeneic leukaemia in primed and unprimed individuals. Nylon-wool fractionation before stimulation with leukaemia cells marginally increased NK-like CMC in the primed patient, but slightly reduced anti-leukaemic CMC. Both types of lytic activity were generated in the nylon-non-adherent fractions, but whether this means that NK-like CMC in primed lymphocytes involves the same population as the anti-leukaemic killer cells remains to be determined. Clearly to accommodate this, "primed" NK-like cells would have to differ qualitatively from those stimulated by leukaemia cells in unprimed individuals.

A number of studies have shown that CMC to autologous and HLA-identical leukaemias can be generated in lymphocytes stimulated with allogeneic lymphoid cells, with or without the appropriate leukaemia (Zarling *et al.*, 1976, 1978; Sondel *et al.*, 1976; Lee & Oliver, 1978). One interpretation of these findings is that the CMC generated by allogeneic cells is NK-like. Zarling's (1978) data using pooled stimulating lymphocytes, without leukaemia cells, suggests that stimulation with a patient's leukaemia may not be mandatory. In addition NK cells may be stimulated by interferon to become cytotoxic to leukaemias (Zarling *et al.*, 1979). In the present study the induction of CMC to allogeneic leukaemias by mixed-cell stimulation did not cause the marked increase in NK-like CMC on CCL which might have been expected had a single type of cytotoxic cell been responsible for both phenomena. However, mixed-cell stimulation might induce qualitatively different NK-like lysis, both on CCL and leukaemia-cells. In the case of the autologous leukaemia, the most plausible explanation is that a specifically antileukaemic cytotoxic population is amplified.

Conditioning factors released in various mixed leucocyte cultures were able to amplify NK-mediated lysis of K562 by unstimulated lymphocytes, and also mildly enhance the killing capacity of leukaemia-cell-stimulated lymphocytes on leukaemia cells. The role of interferon in augmenting NK-like cell-mediated lysis (Trinchieri *et al.*, 1978*b*; Zarling *et al.*, 1979; Moore & Potter, 1980) may be relevant to these observations. Endogenous interferon is induced in mixed lymphocyte cultures (Gifford *et al.*, 1971) and in mixtures of lymphocytes and tumour cells within a few hours of culture (Trinchieri *et al.*, 1978*a*). The high levels of NK-like CMC found in lymphocyte cultures stimulated with leukaemia cells could have been due to either endogenous interferon or factor(s) with similar biological properties. Factor(s) produced by alloantigenic stimulation *in vitro* (killer-cell helper factor) (Orosz & Finke, 1978; Sopori *et al.*, 1978) and exogenous interferon can enhance cytotoxic T-cell activity (Heron *et al.*, 1976) and recent evidence suggests at least one may be inseparable, biochemically, from interferon (Simon *et al.*, 1979).

This work was supported by the Leukaemia Research Fund. The interest of Dr Rodney Harris and assistance of Dr R. Zuhrie are gratefully acknowledged. Mrs Casey gave excellent secretarial services.

### REFERENCES

ANDERSSON, L. C., NILSSON, K. & GAHMBERG, C. G. (1979) K562: A human erythroleukaemic cell line. *Int. J. Cancer*, **23**, 143.

BAKACS, T., GERGELY, P. & KLEIN, E. (1977) Characterisation of cytotoxic human lymphocyte subpopulations: The role of Fc-receptor-carrying cells. *Cell. Immunol.*, **32**, 317.

CALLEWAERT, D. M., KAPLAN, J., PETERSON, W. D. & LIGHTBODY, J. J. (1975) Stimulation in the mixed leukocyte culture and generation of effector cells in cell mediated lympholysis by a human T lymphoblast cell line. *Cell. Immunol.*, **19**, 276.

CALLEWAERT, D. M., KAPLAN, J., PETERSON, W. D. & LIGHTBODY, J. J. (1977) Spontaneous cytotoxicity of human lymphoblast cell lines mediated by normal peripheral blood lymphocytes. 1. Differential susceptibility of T- *versus* B-cell lines. *Cell. Immunol.*, **33**, 11.

CALLEWAERT, D. M., LIGHTBODY, J. J., KAPLAN, J., JAROSZEWSKI, J., PETERSON, W. D. & ROSENBERG, J. C. (1978) Cytotoxicity of human peripheral lymphocytes in cell-mediated lympholysis: Antibody-dependent cell-mediated lympholysis and natural cytotoxicity assays after mixed lymphocyte culture. *J. Immunol.*, **121**, 81.

GIFFORD, G. E., TIBOR, A. & PEAVY, D. L. (1971) Interferon production in mixed lymphocyte cell cultures. *Infect. Immunol.*, **3**, 164.

HARRIS, R., ZUHRIE, S. R., FREEMAN, C. B. & 6 others (1978) Active immunotherapy in acute myelogenous leukaemia and the induction of second and subsequent remissions. *Br. J. Cancer*, **37**, 282.

HERBERMAN, R. B., MCCOY, J. L. & LEVINE, P. H. (1974) Immunological reactions to tumor associated antigens in Burkitt's lymphoma and other lymphomas. *Cancer Res.*, **34**, 1222.

HERBERMAN, R. B. & HOLDEN, H. T. (1978) Natural cell-mediated immunity. *Advances in Cancer Research*, **27**, 305.

HERBERMAN, R. B., DJEU, J. Y., KAY, H. D. & 7 others (1979) Natural killer cells: Characteristics and regulation of activity. *Immunol. Rev.*, **44**, 42.

HERON, I., BERG, K. & CANTELL, K. (1976) Regulatory effect of interferon on T cells *in vitro*. *J. Immunol.*, **117**, 1370.

JENSEN, P. J., AMOS, D. B. & KOREN, H. S. (1979) Depletion of NK by cellular immunoabsorption. *J. Immunol.*, **123**, 1127.

JONDAL, M., SPINA, C. & TARGAN, S. (1978) Human spontaneous killer cells selective for tumour-derived target cells. *Nature*, **272**, 62.

JONDAL, M. & TARGAN, S. (1978) *In vitro* induction of cytotoxic effector cells with spontaneous killer cell specificity. *J. Exp. Med.*, **148**, 1621.

JULIUS, M. H., SIMPSON, E. & HERZENBERG, L. A. (1973) A rapid method for the isolation of functional thymus-derived murine lymphocytes. *Eur. J. Immunol.*, **3**, 645.

KAY, H. D. & SINKOVICS, J. G. (1974) Cytotoxic lymphocytes from normal donors. *Lancet*, ii, 296.

KEDAR, E., RAANEN, Z., KAFKA, I., HOLLAND, J. F., BEKESI, G. J. & WEISS, D. W. (1979) *In vitro* induction of cytotoxic effector cells against human neoplasms. I. Sensitisation conditions and effect of cryopreservation on the induction and expression of cytotoxic responses to allogeneic leukaemia cells. *J. Immunol. Methods*, **28**, 303.

LEE, S. K. & OLIVER, R. T. D. (1978) Autologous leukaemia-specific T cell-mediated lymphocytotoxicity in patients with acute myelogenous leukaemia. *J. Exp. Med.*, **147**, 912.

LOZZIO, C. B. & LOZZIO, B. B. (1975) Human chronic myelogenous leukaemia cell-line with positive Philadelphia chromosome. *Blood*, **45**, 321.

MOORE, M. & POTTER, M. R. (1980) Enhancement of human natural cell-mediated cytotoxicity by interferon. *Br. J. Cancer*, **41**, 378.

NELSON, D. L., BUNDY, B. M. & STROBER, W. (1977) Spontaneous cell-mediated cytotoxicity by human peripheral blood lymphocytes *in vitro*. *J. Immunol.*, **119**, 1401.

OROSZ, C. G. & FINKE, J. H. (1978) Influence of killer assisting factor (KAF) on generation of cytotoxic T cells. *Cell. Immunol.*, **37**, 86.

ORTALDO, J. R. & BONNARD, G. D. (1977) Presence of natural killer cell activity in cytotoxic lymphocytes from MLC–CML reactions. *Fed. Proc.*, **36**, 1325.

PROSS, H. F. & JONDAL, M. (1975) Cytotoxic lymphocytes from normal donors. A functional marker of human non-T lymphocytes. *Clin. Exp. Immunol.*, **21**, 226.

PROSS, H. F., BAINES, M. G. & JONDAL, M. (1977) Spontaneous human lymphocyte-mediated cytotoxicity against tumor target cells. II. Is the complement receptor necessarily present on the killer cells? *Int. J. Cancer*, **20**, 353.

PROSS, H. F. & BAINES, M. G. (1977) Spontaneous human lymphocyte-mediated cytotoxicity against tumor target cells. *Cancer Immunol. Immunother.*, **3**, 75.

ROSENBERG, E. G., MCCOY, J. L., GREEN, S. S. & 4 others (1974) Destruction of human lymphoid tissue-culture cell lines by human peripheral lymphocytes in $^{51}$Cr-release cellular cytotoxicity assays. *J. Natl Cancer Inst.*, **52**, 345.

SAKSELA, E., TIMONEN, T., RANKI, A. & HAYRY, P. (1979) Morphological and functional characterisation of isolated effector cells responsible for human natural killer activity to fetal fibroblasts and cultured cell line targets. *Immunol. Rev.*, **44**, 71.

SANTOLI, D. & KOPROWSKI, H. (1979) Mechanism of activation of human natural killer cells against tumor and virus-infected cells. *Immunol. Rev.*, **44**, 125.

SEELEY, J. K. & GOLUB, S. H. (1978) Studies on cytotoxicity generated in human mixed lymphocyte cultures. I. Time course and target spectrum of several distinct concomitant cytotoxic activities. *J. Immunol.*, **120**, 1415.

SIMON, P. L., FARRAR, J. J. & KIND, P. D. (1979) Biochemical relationship between murine immune interferon and a killer cell helper factor. *J. Immunol.*, **122**, 127.

SONDEL, P. M., O'BRIEN, C., PORTER, L., SCHLOSSMAN, S. F. & CHESS, L. (1976) Cell-mediated destruction of human leukaemic cells by MHC identical lymphocytes: Requirement for a proliferative trigger *in vitro*. *J. Immunol.*, **117**, 2197.

SOPORI, M., BERNSTEIN, A. & BACH, F. H. (1978) *In vitro* sensitisation of thymocytes. Role of H2I region determinants and cell-free mixed leucocyte culture supernates in generation of cytotoxic responses. *J. Exp. Med.*, **148**, 953.

TAYLOR, G. M., HARRIS, R. & FREEMAN, C. B. (1976) Cell-mediated cytotoxicity as a result of immunotherapy in patients with acute myeloid leukaemia. *Br. J. Cancer*, **33**, 137.

TAYLOR, G. M., ZUHRIE, S. R. & HARRIS, R. (1979) Cell-mediated cytotoxicity in relation to active immunotherapy in acute myeloid leukaemia. *Cancer Immunol. Immunother.*, **5**, 263.

TRINCHIERI, G., SANTOLI, D., DEE, R. R. & KNOWLES, B. B. (1978*a*) Anti-viral activity induced by culturing lymphocytes with tumor-derived or virus-transformed cells. Identification of the anti-viral activity as interferon and characterisation of the human effector lymphocyte subpopulations. *J. Exp. Med.*, **147**, 1299.

TRINCHIERI, G. & SANTOLI, D. (1978*b*) Anti-viral activity induced by culturing lymphocytes with tumor-derived or virus-transformed cells. Enhancement of human natural killer cell activity by interferon and antagonistic inhibition of susceptibility of target cells to lysis. *J. Exp. Med.*, **147**, 1314.

WEST, W. H., CANNON, G. B., KAY, H. D., BONNARD, G. D. & HERBERMAN, R. B. (1977) Natural cytotoxic reactivity of human lymphocytes against a myeloid cell line: Characterisation of effector cells. *J. Immunol.*, **118**, 365.

ZARLING, J. M., RAICH, P. C., MCKEOUGH, M. & BACH, F. H. (1976) Generation of cytotoxic lymphocytes *in vitro* against autologous human leukaemia cells. *Nature*, **262**, 691.

ZARLING, J. M., ROBINS, H. I., RAICH, P. C., BACH, F. H. & BACH, M. L. (1978) Generation of cytotoxic T lymphocytes to autologous human leukaemia cells by sensitisation to pooled allogeneic normal cells. *Nature*, **274**, 269.

ZARLING, J. M., ESKRA, L., BORDEN, E. C., HOROSEWICZ, J. & CARTER, W. A. (1979) Activation of human natural killer cells cytotoxic for human leukaemia cells by purified interferon. *J. Immunol.*, **123**, 63.